# Development and validity assessment of a Japanese version of the Exercise Adherence Rating Scale in participants with musculoskeletal disorders

**DOI:** 10.1186/s12955-021-01804-x

**Published:** 2021-06-24

**Authors:** Hiroshi Takasaki, Shota Kawazoe, Takahiro Miki, Hiroki Chiba, Emma Godfrey

**Affiliations:** 1 Department of Physical Therapy, Saitama Prefectural University, Sannomiya 820, Koshigaya, Saitama 343-8540 Japan; 2 Graduate School of Rehabilitation Science, Saitama Prefectural University, Koshigaya, Japan; 3 Department of Psychology, Institute of Psychiatry, Psychology and Neuroscience and Department of Population Health Sciences, School of Population Health and Environmental Sciences, King’s College London, London, UK

**Keywords:** Adherence, Compliance, Construct validity, Cross-cultural adaptation, Physiotherapy, Prescribed exercise, Rasch analysis, Translation

## Abstract

**Background:**

Exercise adherence is important for achieving a long-term effect from musculoskeletal management. The Exercise Adherence Rating Scale (EARS), which was developed in 2017 as a patient reported outcome measure to assess exercise adherence in those with chronic low back pain in the UK, has demonstrated acceptable validity and reliability and is a robust measure of exercise adherence. This study aimed to undertake cross-cultural adaptation of the EARS into Japanese and investigate its structural validity in participants with musculoskeletal disorders.

**Methods:**

The current study was composed of two phases, where a provisional Japanese version of the EARS was developed employing an international guideline for cross-cultural adaptation (Phase A), and structural validity was then evaluated using the Rasch analysis (Phase B). Participants with musculoskeletal disorders who have individualized home exercises prescribed by a physical therapist were recruited.

**Results:**

In Phase A, the pilot testing was conducted twice because the initial testing detected some uncertainty revealed in comments from 17 participants (5 males and 12 females, 18–79 years of age) about which activities and exercises were supposed to be included. We therefore modified the draft by identifying a person who prescribed/recommended activities and exercises as per the Working Alliance Inventory. The second pilot testing using this draft recruited 30 participants (6 males and 24 females, 18–79 years of age), who provided no further comments, demonstrating the Japanese version of the EARS (EARS-J) had been successfully developed. In Phase B, data from 200 participants who completed the EARS-J (63 males and 127 females, mean ± SD of age = 53.6 ± 17.0) were analyzed using the Andrich's Rating Scale Model. Rasch statics indicated unidimensionality of the six items of the EARS-J. The Cronbach *α* was 0.77. Substantial ceiling effect (21.0%) was observed, with no floor effect (0.5%).

**Conclusions:**

A Japanese version of the EARS has been developed, which demonstrated acceptable structural validity with the evidence of unidimensionality in the Rasch analysis in Japanese people with musculoskeletal disorders who were prescribed individualized home exercises. However, there was a substantial ceiling effect and further studies are required to comprehensively establish validity and reliability of the EARS-J.

**Supplementary Information:**

The online version contains supplementary material available at 10.1186/s12955-021-01804-x.

## Background

The World Health Organization defines adherence as the extent to which a person’s behavior corresponds with agreed recommendations from a healthcare provider [1] Frost et al. [2] refined this for rehabilitation and proposed adherence be defined as the extent to which individuals undertake a prescribed behavior accurately and at the agreed frequency, intensity and duration. Exercise adherence is important to achieve a long-term effect from musculoskeletal management [3]. Systematic reviews have stated that exercise adherence strategy is a future research priority and standardized and validated measures of exercise adherence are warranted [4, 5].

In response to this, the Exercise Adherence Rating Scale (EARS) was developed in 2017 as a patient reported outcome measure (PROM) to assess exercise adherence in those with chronic low back pain (LBP) in the UK [6]. The EARS is composed of six items assessed via a 5-point Likert scale, whose possible sum scores ranges from 0 to 24. The EARS has demonstrated structural validity that was shown as a one-factor solution explaining a total of 71% of the variance in adherence to exercise in the exploratory factor analysis in people with chronic LBP [6]. The EARS also demonstrated good internal consistency (Cronbach's α = 0.81) and test–retest reliability (intra-class correlation coefficient [ICC] = 0.97) in people with chronic LBP [6]. Cross-cultural adaptation has been initiated in several populations. The Brazilian Portuguese version has been developed in people with non-specific chronic LBP, showing one factor solution in the confirmatory factor analysis and good test–retest reliability (ICC = 0.91) [7]. The Nepali version has been developed in people with pre-diabetes or confirmed diagnosis of any disease, showing a one-factor solution in the exploratory factor analysis [8]. However, a valid Japanese version of the EARS has not yet been established. Further, the structural validity has not been tested with the more robust statistical method of Rasch analysis and in a broader population of people with musculoskeletal disorders.

The aim of the current study was to undertake cross-cultural adaptation of the EARS into Japanese and investigate its structural validity in participants with musculoskeletal disorders.

## Methods

### Overall design

The current study was composed of two phases (Phase A and B). In Phase A, a provisional Japanese version of the EARS (EARS-J) was developed through four stages using an international guideline for cross-cultural adaptation [9]. In Phase B, structural validity and internal consistency were evaluated using Rasch analysis. This study has been granted approval by a human research ethics committee in the Saitama Prefectural University (No. 19057). The manuscript has been prepared with a reference of the STROBE (Strengthening The Reporting of OBservational Studies in Epidemiology) Checklist.

### Phase A: Participants

Using convenience sampling, participants with musculoskeletal disorders referred to outpatient physical therapy were included from September 2018 to December 2018. Participant’s inclusion criteria were (1) > 18 years of age, (2) having individualized home exercises prescribed as management for musculoskeletal disorders by a physical therapist, and (3) no history of diagnosed cognitive or neurological disorders. Participants were recruited from a primary care center in Japan (Minami Shinjuku Orthopedic Clinic, Tokyo, Japan). Written informed consent was obtained from each participant before data collection.

### Phase A: Process of cross-cultural adaptation of the EARS

First, approval for cross-cultural adaptation of the EARS was obtained from the developer (EG). In Stage I of the initial translation, forward-translation was undertaken independently by two translators whose first language was Japanese. One translator was a physical therapist (HT), who understood the concept of the EARS and knew about the original study [6]. Another translator was an English scholar, who was unaware of the EARS and the original study [6]. In Stage II of the synthesis of the translations, a combined Japanese version (Draft 1) was developed through discussions between an author (SK) and the two forward-translations. Subsequently, in Stage III of the back translation, Draft 1 was translated into English independently by translators whose first language was English. The two translators were physical therapists, who were unfamiliar with the EARS and the original study [6]. In Stage IV, an expert committee (n = 5) including all translators and authors (HT and SK) reviewed the original EARS and all the translations, and reached a consensus on any discrepancies. Further, the developer (EG) then assessed two backward-translations of Draft 1 and confirmed consistency of meanings between the original EARS and Draft 1. In Stage V, testing the prefinal version, the final draft was assessed through pilot testing with 30 participants using a paper–pencil survey.

### Phase B: Participants

Using the same eligibility criteria and sampling method as Phase A, participants (musculoskeletal disorders referred to outpatient physical therapy) were included from three primary care centers in Japan (Minami Shinjuku Orthopedic Clinic, Tokyo; Secomedic Hospital, Chiba; and Sapporo Maruyama Orthopedic Hospital, Sapporo) from December 2018 to December 2019. Data were collected with an anonymous online survey using SurveyMonkey (SurveyMonkey, San Mateo, CA, USA) and consent was obtained by answering the survey.

### Phase B: Process of data analysis

Two-hundred participants were recruited as a sample size of 200 was suggested as necessary in order to run Rach analysis [10]. The participants completed the EARS-J, the 4-item pain intensity measure (P4) [11] and SF-12v2® Health Survey [12] and provided their demographic details, as well as predominant symptom location and symptom duration. The EARS-J is composed of six items with 5-point Likert scale (0 = completely agree, 4 = completely disagree). Items 2, 3 and 5 are negatively worded questions. Therefore, higher sum scores (0–24) indicate greater exercise adherence by reversing scores of Items 1, 4, and 6. The P4 is a measure of pain intensity. The P4 includes four 11-point numerical rating scales for pain intensity over the last two days, where higher sum scores (0–40) indicate higher pain intensity [13]. The construct validity and test–retest reliability (ICC [95% confidence intervals] = 0.78 [0.72–0.83]) have been established [13]. The SF-12v2® Health Survey is a shorter version of the SF-36v2® Health Survey and an established measure of physical and mental health using the physical component summary score, and the mental component summary score, respectively. The value of 50 indicates Japanese normal score, and a higher score indicates better health condition.

Rasch analysis was conducted using the Andrich's Rating Scale Model with the Winsteps version 3.93 (Winsteps.com, Beaverton, OR, USA). Unidimensionality of the EARS-J was assessed using criteria in previous studies [14–17]. Briefly, unidimensionality was considered when all the following criteria were satisfied: (1) ≥ 60% of the raw variance was explained by the measure, (2) the eigenvalue of < 2 in the first contrast, and 3) infit/outfit mean-square (MnSq) statistics of 0.6–1.4 and their standard Z-values of − 2 to 2. An item with a MnSq of far greater than 1.4 and a standard Z-value of far greater than 2 is considered to have a different construct from other items. Differential Item Functioning (DIF) in gender was also assessed using the Rasch-Welch t-test, where a statistical significance level was set < 0.05.

Response distribution was also assessed by visualizing an item-person map and assessing floor and ceiling effects. A threshold of 15% was used for the assessment of floor and ceiling effects [14, 15]. Further, a conversion table from raw total score to the 0–100 Rasch score was also created to allow the use of the 0–100 Rasch score, which is considered to be more normally distributed than the use of the raw total score (0 to 24). Internal consistency was assessed as Cronbach’s *α*, where α of greater than 0.7 was considered acceptable in the current study [18].

## Results

### Phase A

**Fig. 1:**
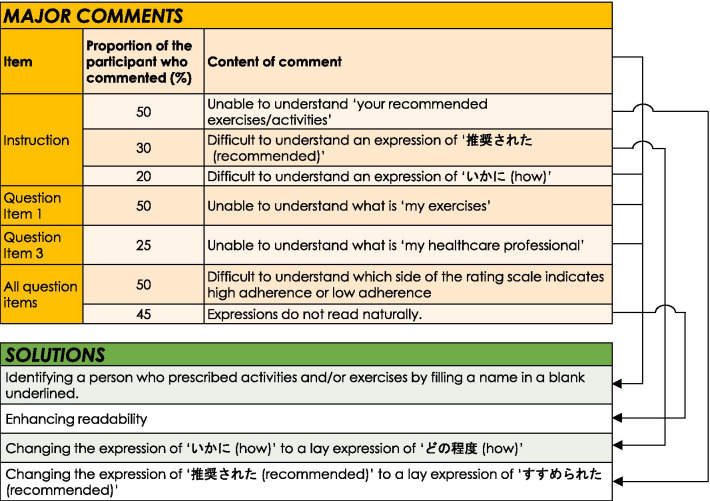
Major comments provided by ≥ 20% of the 17 participants and solutions in Phase A

During the pilot testing, some concerns were detected when 17 of the participants (5 males and 12 females, age = 18–79 years of age, mean ± SD of age = 54 ± 17 years, and mean ± SD of the P4 for pain intensity = 14 ± 9) read Draft 1 and provided comments on readability and any confusion about meanings in the instructions, item descriptions, scoring method, and recall period. Therefore, a reconciliation meeting was held between the five members of the expert committee, which included advice from the developer (EG). Consequently, another combined version was developed (Draft 2), where Fig. 1 presents major comments provided by ≥ 20% of the participants and solutions. In response to a common comment on uncertainty about which activities and exercises are supposed to be included, we modified Draft 2 by identifying a person who prescribed/recommended activities and exercises as per the Working Alliance Inventory [19]. Negatively worded questions were maintained as per advice from the developer. Another subset of two translators whose first language was English, and were not medical professionals, translated Draft 2 into English with no previous knowledge of the EARS or the original study [6]. The developer assessed the two backward-translations of Draft 2 and confirmed consistency of meanings between the original EARS and the Draft 2. Consequently, all comments from the participants were successfully resolved in Draft 2. Subsequent pilot testing with another 30 participants (6 males and 24 females, age = 18–79 years of age, mean ± SD of age = 50 ± 19 years, and mean ± SD of the P4 = 17 ± 9) completed Draft 2 while measuring time to complete, and then provided comments on readability and any confusion over meanings as in the first round of pilot testing. There were no further comments on Draft 2. Mean ± SD time for completion of the Draft 2 was 80 ± 36 s. Consequently, Draft 2 became the EARS-J (Additional file 1) and was approved by the developer.

### Phase B

**Fig. 2:**
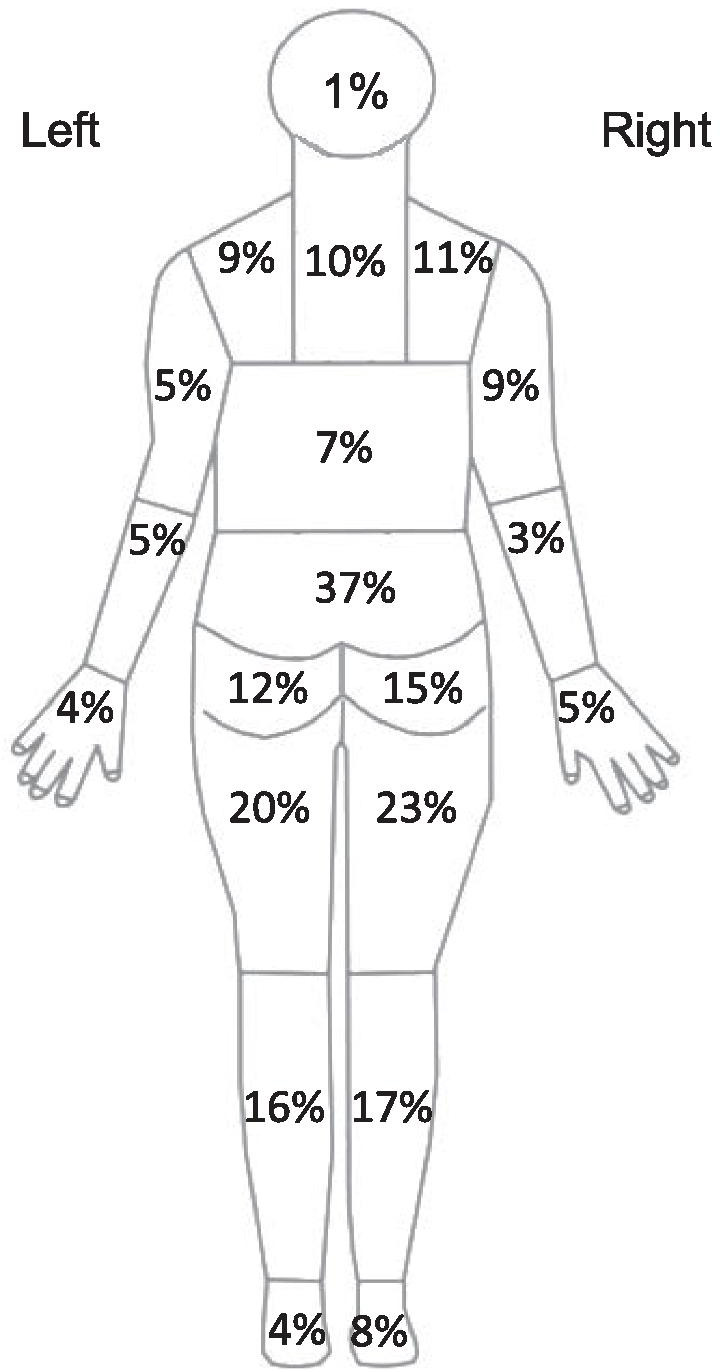
Symptom distributions of the sample in Phase B. n = 184

Table 1 presents demographic characteristics of the 200 participants in Phase 2. Figure 2 presents symptom distributions. Two participants missed two data points of the EARS-J and missing data was 4 points out of the 1200 points (6 items × 200 participants) in the EARS-J. No data imputation technique was undertaken.

**Table 1:** Demographic summary of the 200 participants in Phase 2

Age (year), mean (SD), (n = 188)	53.6 (17.0)
Gender (n = 190)	
Male, n (%)	63 (33.2)
Female, n (%)	127 (66.8)
Symptom duration (month), mean (SD), (n = 168)	17.8 (36.9)
4-item pain intensity measure (0–40), mean (SD), (n = 200)	11.0 (8.6)
SF-12v2® Health Survey Physical Component Summary score, mean (SD), (n = 183)	43.6 (4.3)
SF-12v2® Health Survey Mental Component Summary score, mean (SD), (n = 183)	49.6 (1.7)

SD, standard deviations

In the EARS-J, the eigenvalue in the first contrast was 1.92 and 61.2% of the raw variance was explained by the measure. Table 2 presents fit statistics in the EARS-J. These indicated unidimensionality of the EARS-J.

**Table 2:** Fit statistics in the Japanese version of the Exercise Adherence Rating Scale

Item description	Measure	SE	Infit MnSq	Infit Zstd	Outfit MsSq	Outfit Zstd
Item 3. I do less exercise than recommended by my healthcare professional	1.45	0.08	0.88	− 1.1	0.83	− 1.0
Item 2: I forget to do my exercises	1.14	0.08	1.17	1.6	1.26	2.2
Item 5.* I don’t get around to doing my exercises	− 0.16	0.09	1.17	1.5	0.95	− 0.3
Item 6.* I do most, or all, of my exercises	− 0.66	0.10	1.03	0.3	0.89	− 0.5
Item 4.* I fit my exercises into my regular routine	− 0.82	0.11	0.74	− 2.0	0.77	− 1.0
Item 1.* I do my exercises as often as recommended	− 0.95	0.11	1.01	0.1	1.14	0.6

SE, standard error of measurement; MnSq, mean square; Zstd, standardized Z-value

*Using reverse scores

**Fig. 3:**
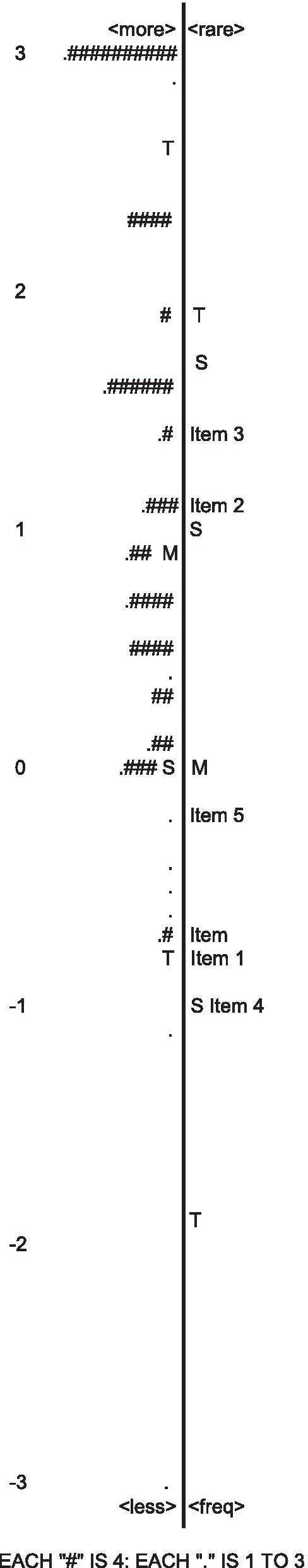
Item-person map

The Rasch item-person map (Fig. 3) showed that the mean of person ability was greater than the mean of item difficulty and there was no item covering high person ability such as the ones around logit 3. The map indicated that items (for example, Items 1, 4, and 6) were disagreed with by most participants in this cohort study, indicating that most participants chose responses of high adherence. Substantial ceiling effect (21.0%) was observed, with no floor effect (0.5%). Table 3 presents a conversion table from raw total score to the 0–100 Rasch score.

**Table 3:** Conversion table from raw total score to the 0–100 Rasch score

Raw total score	0–100 Rasch score
0	0
1	14
2	22
3	27
4	30
5	33
6	36
7	39
8	41
9	43
10	45
11	48
12	50
13	52
14	54
15	57
16	59
17	62
18	65
19	68
20	71
21	75
22	80
23	87
24	100

The DIF analysis (Table 4) showed no statistically significant DIF in gender (all *p* values ≥ 0.05). The Cronbach α was 0.77, indicating acceptable internal consistency.

**Table 4:** Results of the differential item functioning (DIF)

Item	Class A	DIF	DIF SE	Class B	DIF	DIF SE	DIF Contrast	df	Welch t	*p*
1	Female	− 0.76	0.14	Male	− 1	0.19	0.24	99	1.02	0.3124
2	Female	1.14	0.1	Male	1.32	0.14	− 0.18	99	− 1.01	0.3143
3	Female	1.39	0.1	Male	1.59	0.15	− 0.2	97	− 1.09	0.2789
4	Female	− 0.79	0.14	Male	− 1.3	0.22	0.52	87	1.99	0.05
5	Female	− 0.12	0.11	Male	− 0.21	0.15	0.09	103	0.46	0.6478
6	Female	− 0.84	0.14	Male	− 0.66	0.17	− 0.19	112	− 0.84	0.4003

Male = 63 participants, Female = 127 participants

SE, standard error of measurement; df, degree of freedom

## Discussion

In the cross-cultural adaptation process of the EARS into Japanese, there was a common comment about uncertainty over which activities and exercises were supposed to be included. This comment corresponds with a finding reported in a previous study with musculoskeletal disorders using the original English version of the EARS [20]. Instructions were improved by specifying who had prescribed physical activity and exercises as per the Working Alliance Inventory [9]. The original EARS reported the content validity through a 4-stage process (focus group, expert advice, consideration of the literature, and feedback from 20 patient) in people with chronic LBP [6]. However, a subsequent study [20] employing people with persistent musculoskeletal disorders to test face validity reported that the EARS questionnaire is understandable and has good face validity, but that instructions needed to be modified to make it clear that the questionnaire referred to prescribed exercise or specific physical activity recommendations.

Rasch analysis demonstrated that the EARS-J had acceptable unidimensionality and no substantial concerns in the DIF analysis, indicating no clear response pattern due to gender. These findings support structural validity with a one factor solution explaining a total of 71% of the variance using the exploratory factor analysis and internal consistency (Cronbach’s α = 0.81) observed in the original EARS [6]. We also found that the mean time for completion of the EARS-J was 80 s. A median of 10 min is considered to be feasible in a web survey [21]. Thus, the EARS-J is expected to be used together with other measures. These findings provide us with a promising foundation for further investigation of validity and reliability in the EARS-J to establish an easily administered, valid and reliable tool to understand exercise adherence.

Within primary psychometric properties proposed in COSMIN [22], the results with the original EARS demonstrated internal consistency (Cronbach’s α = 0.81), test–retest reliability for 3 weeks (ICC = 0.97), and structural validity using exploratory factor analysis [6]. The remaining primary psychometric properties to be tested in the future included measurement errors, criterion related validity, and responsiveness. To investigate measurement errors, care should be taken about the population used in the sample, for example, those with more stable symptoms and consistent management strategies. To investigate criterion related validity, attention needs to be given to blinding of purpose, participant, and outcome. For example, the criterion related validity of the EARS could be examined with the correlation between EARS scores for a home strengthening knee exercise and its adherence measured using an accelerometer concealed in an ankle cuff weight as investigated by Nicolson et al. [23] using the self-reported measures of a paper exercise diary and self-reported adherence on an 11-point numerical rating scale. To investigate responsiveness, care needs to be taken when controlling confounding internal and external factors, such as patient empowerment and healthcare providers’ patient engagement approaches.

## Limitation

The Rasch item-person map demonstrated that the mean of person ability was greater than the mean of item difficulty and the distribution of item difficulty did not cover that of person ability. These findings would reflect a ceiling effect of the EARS-J and a potential limitation of the current study, which could be associated with the population of interest in the current study and convenience sampling. The current study included those who had been prescribed home exercise by a physical therapist; however, musculoskeletal management can also be conducted with group exercises. Participation in the study was voluntary and so there could have been self-selection bias, where those with low exercise adherence might not have wanted to have physical therapy and/or participate in the study, and self-presentation bias, where those with high exercise adherence might have chosen to participate in the study. Furthermore, we did not control for and investigate time from the prescription of certain exercises to answering the EARS. This factor may have contributed to the biased distribution of the EARS scores. Thus, further investigations using more robust sampling methods and wider populations with musculoskeletal disorders are required to comprehensively establish the validity and reliability of the EARS-J.

## Conclusions

A Japanese version of the EARS was developed through a cross-cultural adaptation process. The Japanese version demonstrated acceptable structural validity with the evidence of unidimensionality in the Rasch analysis in Japanese people with musculoskeletal disorders who were prescribed individualized home exercises. However, there was a substantial ceiling effect and further studies using more robust sampling methods and wider populations with musculoskeletal disorders are required to comprehensively establish the validity and reliability of the EARS-J.

## Supplementary Information

**Additional file 1**. Japanese version of the Exercise Adherence Rating Scale. Modified from [6] under a CC BY license, printed with permission from ELSEVIER, original copyright 2017.

## Data Availability

The datasets used and/or analyzed during the current study are available from the corresponding author on reasonable request.

## Abbreviations

EARS: Exercise Adherence Rating Scale
PROM: Patient reported outcome measure
LBP: Low back pain
ICC: Intra-class correlation coefficient
EARS-J: Japanese version of the EARS
