# Supplementary material for: Development and validity assessment of a Japanese version of the Exercise Adherence Rating Scale in participants with musculoskeletal disorders
Source: Health Qual Life Outcomes. 2021 Jun 24;19:169. doi: 10.1186/s12955-021-01804-x (PMC8223386; doi:10.1186/s12955-021-01804-x)
Supplement: Supplementary file 1 — Additional file 1. Japanese version of the Exercise Adherence Rating Scale. Modified from [6] under a CC BY license, printed with permission from ELSEVIER, original copyright 2017. [file 12955_2021_1804_MOESM1_ESM.docx]

**運動遵守評価尺度（EARS）**

**アンケート実施者への注意事項**

アンケート実施者は、質問文に設けられているXXX(下線部)に運動を処方した者の名前を記入して、回答者が誰に運動を処方されたのかをわかるようにすること。なお、上記下線部に名前を入力すると、自動で次のページの下線部に名前が入ります。

以下の6つの記述は、治療としてすすめられた運動や活動を、あなたがどの程度アドバイス通りに行なっているかを評価するものです。あなたの状況として最も適切な□にチェックマーク（✓）を付けて下さい。

1. **私は、**XXX**にすすめられた運動や活動をアドバイスされた頻度でしている。**

*全く 全く*

*そうである 　　　 そうではない*

| *0*  ☐ | *1*  ☐ | *2*  ☐ | *3*  ☐ | *4*  ☐ |
| --- | --- | --- | --- | --- |

1. **私は、**XXX**にすすめられた運動や活動をし忘れる。**

*全く 全く*

*そうである 　　　 そうではない*

| *0*  ☐ | *1*  ☐ | *2*  ☐ | *3*  ☐ | *4*  ☐ |
| --- | --- | --- | --- | --- |

1. **私は、**XXX**にすすめられたよりも少ない運動や活動をしている。**

*全く 全く*

*そうである 　　　 そうではない*

| *0*  ☐ | *1*  ☐ | *2*  ☐ | *3*  ☐ | *4*  ☐ |
| --- | --- | --- | --- | --- |

1. **私は、**XXX**にすすめられた運動や活動を日課にしている。**

*全く 全く*

*そうである 　　　 そうではない*

| *0*  ☐ | *1*  ☐ | *2*  ☐ | *3*  ☐ | *4*  ☐ |
| --- | --- | --- | --- | --- |

1. **私は、**XXX**にすすめられた運動や活動にとりかかっていない。**

*全く 全く*

*そうである 　　　 そうではない*

| *0*  ☐ | *1*  ☐ | *2*  ☐ | *3*  ☐ | *4*  ☐ |
| --- | --- | --- | --- | --- |

1. **私は、**XXX**にすすめられた全ての運動や活動をしている。**

*全く 全く*

*そうである 　　　 そうではない*

| *0*  ☐ | *1*  ☐ | *2*  ☐ | *3*  ☐ | *4*  ☐ |
| --- | --- | --- | --- | --- |
